# Supplementary material for: Epigenetic identification of mitogen-activated protein kinase 10 as a functional tumor suppressor and clinical significance for hepatocellular carcinoma
Source: PeerJ. 2021 Feb 2;9:e10810. doi: 10.7717/peerj.10810 (PMC7863782; doi:10.7717/peerj.10810)
Supplement: Supplemental Information 1 [file peerj-09-10810-s001.zip › 3.pdf]

MAPK10 — MSP

(44)

22/11/2016

MAPK10 MSP (A+T)

primes:

m { G03 (plate 7) — m_1
G04 (plate 7) — m_2 }

U { G05 (plate 7) — U_1
G06 (plate 7) — U_2 }

Samples:
① Hep3B
② Hep3B (A+T)
③ HepG2
④ HepG2 (A+T)
⑤ SNU 387 · ?
⑥ SNU 387 (A+T)
⑦ positive : Ycc-B1
⑧ -ve

m : 60°C 40 cycles
U : 58°C 40 cycles
2% Gel. 2.5μl syber safe
① 150V 1 min
② 130V 18 min

MAPK10.
RT-PCR.
全部肝Ca的 13个
加上全部的 A+T的
一起做.
阳性对照为: Normal liver.

MAPK10.
MSP. [illegible]

GAPDH
的引物来做.
RT-PCR

MSP for cell lines (using less Taq-Gold/1x) (since July 06') *July 26, 2006, Tao/CEL/CUHK*

95°C/10 min, 40 cycles (94°C/30", °C/30", 72°C/30"), 72°C/5 min	For 25 μl reaction		Final Conc.
10 x **PCR buffer II** (PE)	2.5 μl		1x
25mM $MgCl_2$ (PE)	2 μl		2 mM
dNTP (2.5mM each)	2 μl		0.2 mM
5' primer (10μM)	1.5 μl		0.6 μM
3' primer (10μM)	1.5 μl		0.6 μM
**Taq-Gold** (5U/μl) (PE)	0.125 μl		1.25U / 50μl (1x)
dd H_2O	14.375 μl		
Bisulfited DNA	1 μl		0.1μg /100μl
Total:	*25 μl*		

*(For DNA samples bisulfited before 3/2003', use **0.5ul/25ul**; for DNA samples bisulfited after 3/2003', use **1ul/25ul**)
