# Supplementary material for: Epigenetic identification of mitogen-activated protein kinase 10 as a functional tumor suppressor and clinical significance for hepatocellular carcinoma
Source: PeerJ. 2021 Feb 2;9:e10810. doi: 10.7717/peerj.10810 (PMC7863782; doi:10.7717/peerj.10810)
Supplement: Supplemental Information 1 [file peerj-09-10810-s001.zip › 4.pdf]

MAPK10 — RT PCR and MSP (cell lines)

Nov. 6. 2010 (46)

Primes:

RT-PCR (for MAPK10): { F3 - F11 / R3 - F12 } 248 bp — GAPDH { 3334 - GAPDH33 / R55 - 3335 - GAPDH55 }

MSP: m { m1 - G03 / m2 - G04 } 160 bp — U { U1 - G05 / U2 - G06 } 163 bp

Samples: ① Hep3B ② HepG2 ③ huH1 ④ huH4 ⑤ huH6
⑥ huH7 ⑦ Mahlavu ⑧ PLC/PRF/5 ⑨ SNU387
⑩ SNU398 ⑪ SNU423 ⑫ SNU449 ⑬ SNU475
⑭ normal control: normal liver for RT-PCR
YCC-B1 for MSP
⑮ negative control (-ve)

RT-PCR
55°C 32 cycles (for GAPDH 23 cycles)

RT-PCR using Promega GoTaq *July 16, 2007/CUHK/Tao*

	*95ºC/2', **32** cycles (95°C/30'', °C/30'', 72°C/30''), 72°C/3'*	For 50 µl	For 12.5 µl	×16	Final concentrat.
1:	5 x **Flexi** buffer	8 µl	2 µl	32	0.8x
2:	***5' primer*** (10µM)	3 µl	0.75 µl	12	0.6 µM
3:	***3' primer*** (10µM)	3 µl	0.75 µl	12	0.6 µM
4:	**Promega GoTaq** (5U/µl)	0.25 µl	0.0625 µl	1	1.25U/50 µl
5:	RT product	10 µl	2.5 µl	→ 2.5	about 0.5 µg/50 µl
6:	$MgCl_2$ (25 mM)	2 µl	0.5 µl	8	2 mM
7:	dd H_2O	23.75 µl	5.9375 µl	95	
	Total volume	***50 µl***	***12.5 µl***		

(For *GAPDH*, use 23 cycles)

MSP
m: 58°C 40 cycles — U: 58°C 40 cycles

MSP for cell lines (using less Taq-Gold/1x) (since July 06') *July 26, 2006, Tao/CEL/CUHK*

	95°C/10 min, 40 cycles (94°C/30", °C/30", 72°C/30"), 72°C/5 min	*For 25 µl reaction*	12.5 µl	16	20	*Final Conc.*
						1x
1:	**10 x PCR buffer II** (PE)	2.5 µl	1.25	20		2 mM
2:	25mM $MgCl_2$ (PE)	2 µl	1	16		0.2 mM
3:	dNTP (2.5mM each)	2 µl	1	16		0.6 µM
4:	*5' primer* (10µM)	1.5 µl	0.75	12		0.6 µM
5:	*3' primer* (10µM)	1.5 µl	0.75	12		1.25U / 50µl (1x)
6:	**Taq-Gold** (5U/µl) (PE)	0.125 µl	0.0625	1		
7:	*dd* H_2O	14.375 µl	7.1875	115		0.1µg /100µl
8:	*Bisulfited DNA*	1 µl	0.5	→ 0.5		
	Total:	***25 µl***				

*(For DNA samples bisulfited before 3/2003', use **0.5ul/25ul**; for DNA samples bisulfited after 3/2003', use **1ul/25ul**)

2% Gel ① 150V 1min ② 130V 18min
