# Supplementary material for: Epigenetic identification of mitogen-activated protein kinase 10 as a functional tumor suppressor and clinical significance for hepatocellular carcinoma
Source: PeerJ. 2021 Feb 2;9:e10810. doi: 10.7717/peerj.10810 (PMC7863782; doi:10.7717/peerj.10810)
Supplement: Supplemental Information 1 [file peerj-09-10810-s001.zip › 5.pdf]

MAPK 10

RT PCR for MAPK 10 c cell lines and A+T)

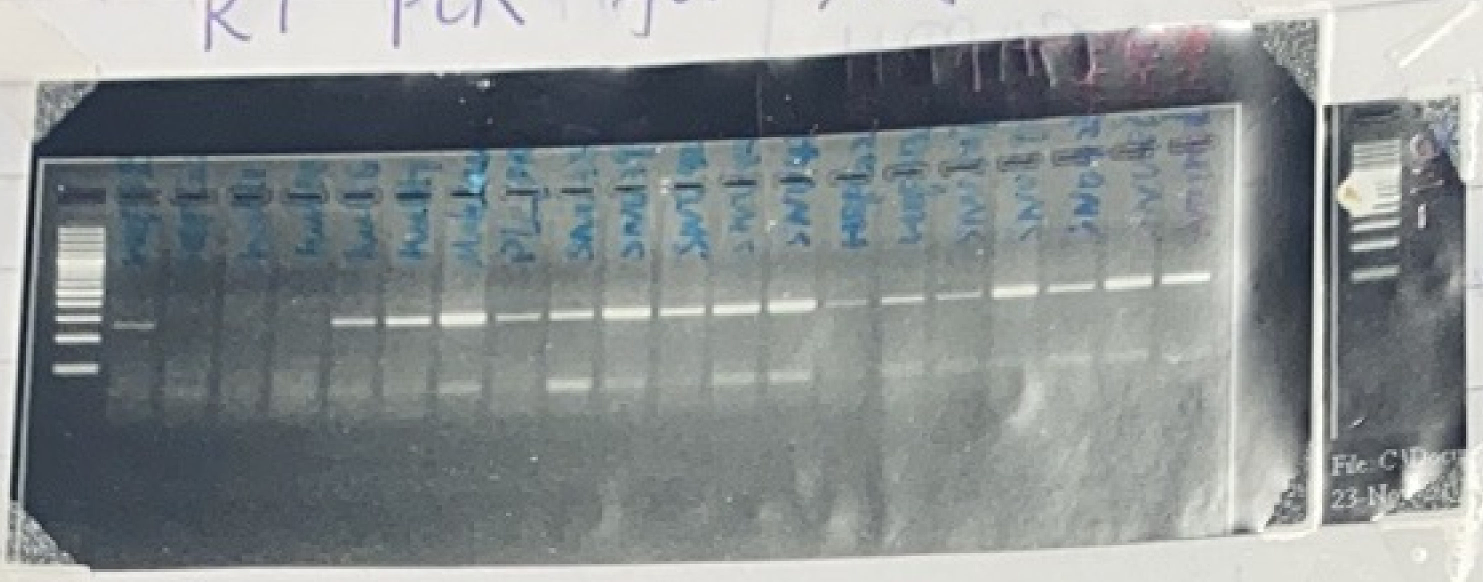

55°C 32 cycles
2% gel

RT PCR for GAPDH

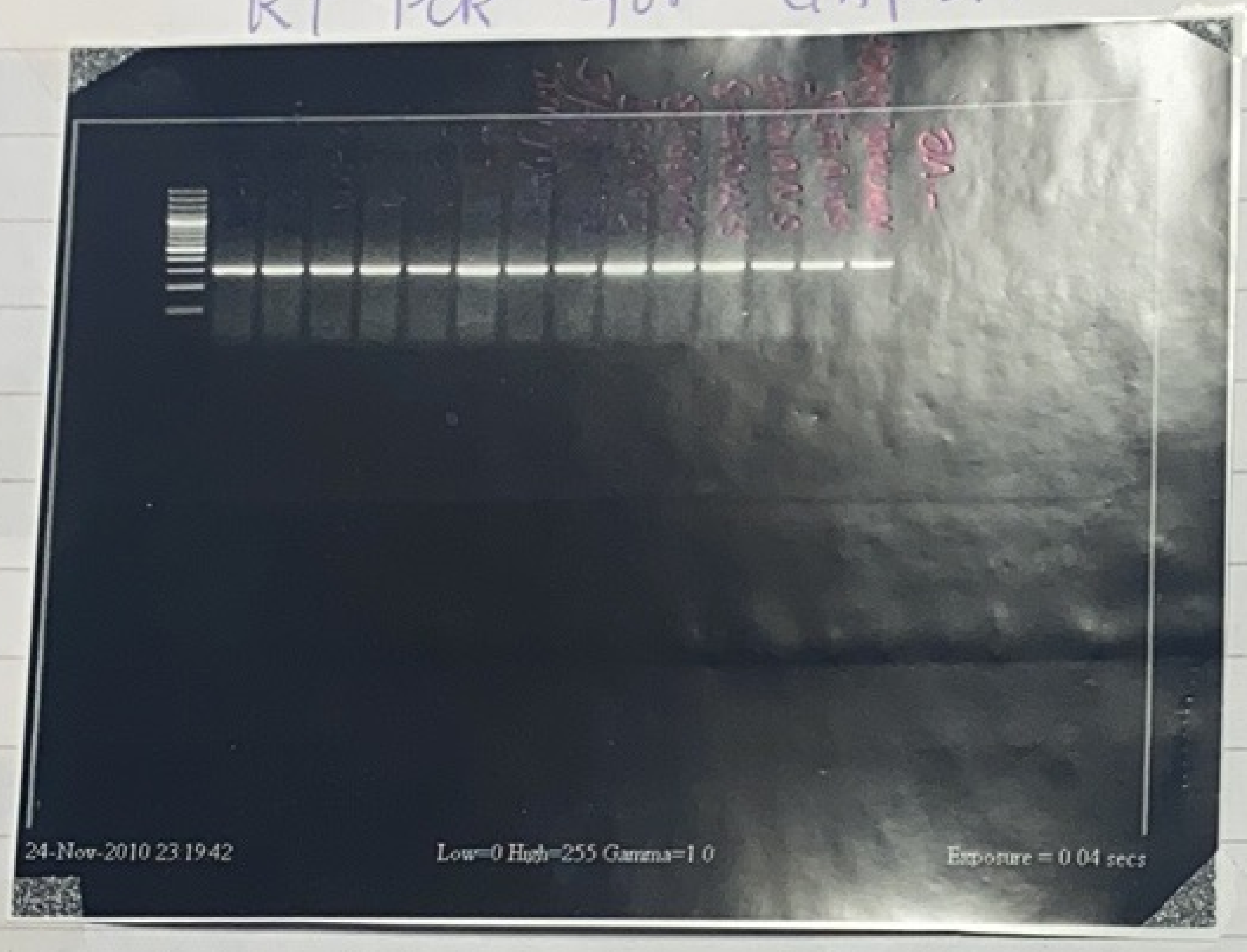

55°C 23 cycles
2% gel

m

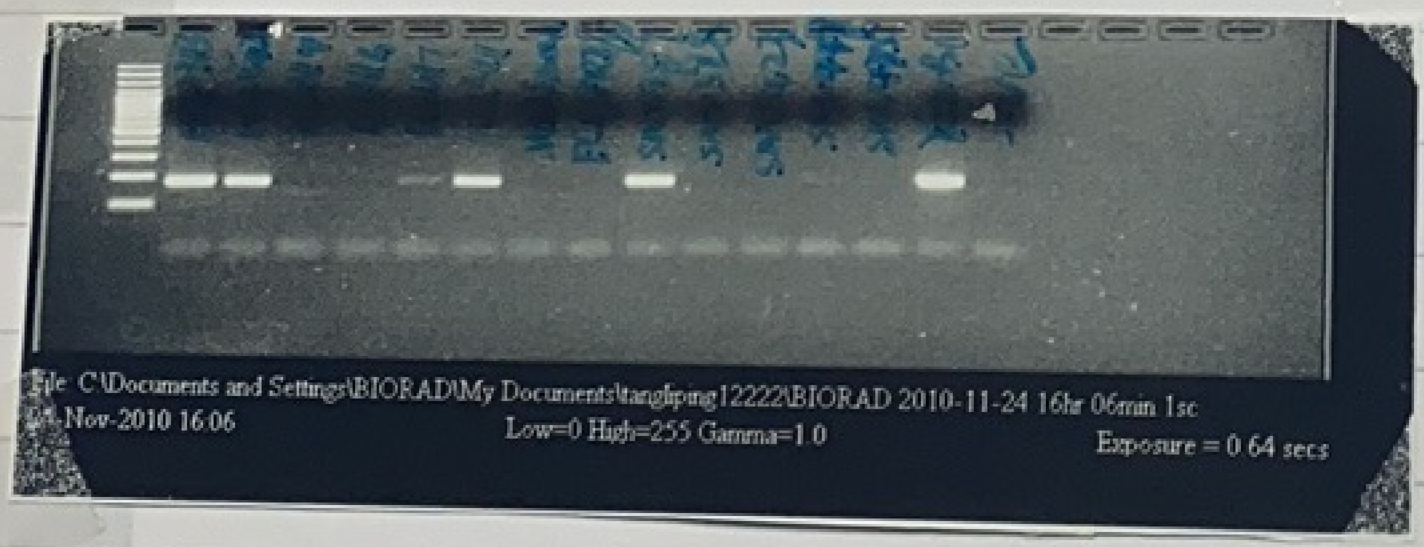

58°C 40 cycles
2% gel

U

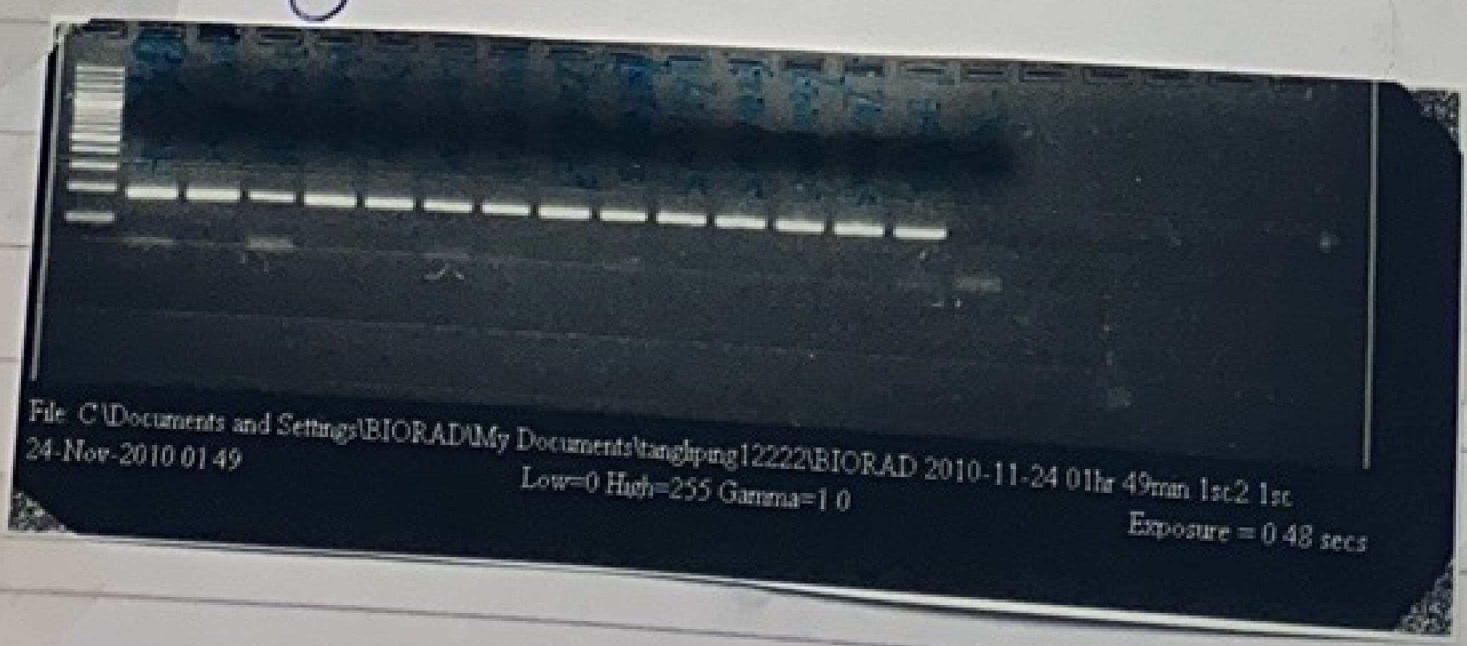

58°C 40 cycles
2% gel
