# Supplementary material for: Epigenetic identification of mitogen-activated protein kinase 10 as a functional tumor suppressor and clinical significance for hepatocellular carcinoma
Source: PeerJ. 2021 Feb 2;9:e10810. doi: 10.7717/peerj.10810 (PMC7863782; doi:10.7717/peerj.10810)
Supplement: Supplemental Information 1 [file peerj-09-10810-s001.zip › 6.pdf]

MAPK10 — RT PCR and MSP (A+T) 24/11/2010 (45)

primers:

RT-PCR: MAPK10 { F3 — F11 ; R3 — F12 }
GAPDH { GAPDH 33 — 3334 ; GAPDH 55 — 3335 }

MSP: m { m1 — G03 ; m2 — G04 }
U { U1 — G05 ; U2 — G06 }

samples:

1. Hep3B
2. Hep3B (A+T)
3. HepG2
4. HepG2 (A+T)
5. SNU 387
6. SNU 387 (A+T)
7. positive (normal liver for RT PCR. YCC-B1 for msp)
8. -ve

RT-PCR:
55°C 32 cycles (GAPDH: 55°C 23 cycles)

RT-PCR using Promega GoTaq *July 16, 2007/CUHK/Tao*

	95°C/2', 32 cycles (95°C/30'', °C/30'', 72°C/30''), 72°C/3'	For 50 µl	For 12.5 µl	×8	Final concentrat.
1:	5 x **Flexi** buffer	8 µl	2 µl	16	0.8x
2:	***5' primer*** (10µM)	3 µl	0.75 µl	6	0.6 µM
3:	***3' primer*** (10µM)	3 µl	0.75 µl	6	0.6 µM
4:	**Promega GoTaq** (5U/µl)	0.25 µl	0.0625 µl	0.5	1.25U/50 µl
5:	RT product	10 µl	2.5 µl	→ 0.5	about 0.5 µg/50 µl
6:	$MgCl_2$ (25 mM)	2 µl	0.5 µl	4	2 mM
7:	dd H_2O	23.75 µl	5.9375 µl	47.5	
	Total volume	***50 µl***	***12.5 µl***		

MSP: m: 60°C 40 cycles   U: 58°C 40 cycles

MSP for cell lines (using less Taq-Gold/1x) (since July 06') *July 26, 2006, Tao/CEL/CUHK*

	95°C/10 min, 40 cycles (94°C/30", °C/30", 72°C/30"), 72°C/5 min	For 25 µl reaction	12.5 µl	×8	Final Conc.
1:	10 x **PCR buffer II** (PE)	2.5 µl	1.25	10	1x
2:	25mM $MgCl_2$ (PE)	2 µl	1	8	2 mM
3:	dNTP (2.5mM each)	2 µl	1	8	0.2 mM
4:	***5' primer*** (10µM)	1.5 µl	0.75	6	0.6 µM
5:	***3' primer*** (10µM)	1.5 µl	0.75	6	0.6 µM
6:	**Taq-Gold** (5U/µl) **(PE)**	0.125 µl	0.0625	0.5	1.25U / 50µl (1x)
7:	*dd* H_2O	14.375 µl	7.1875	57.5	
8:	*Bisulfited DNA*	1 µl	0.5	→ 0.5	0.1µg /100µl
	Total:	***25 µl***			
