# Supplementary material for: Epigenetic identification of mitogen-activated protein kinase 10 as a functional tumor suppressor and clinical significance for hepatocellular carcinoma
Source: PeerJ. 2021 Feb 2;9:e10810. doi: 10.7717/peerj.10810 (PMC7863782; doi:10.7717/peerj.10810)
Supplement: Supplemental Information 1 [file peerj-09-10810-s001.zip › Original pictures.docx]

The original western blot images

Hep3B HepG2

Vector MAPK10（1） MAPK10（2） Vector MAPK10

makp10


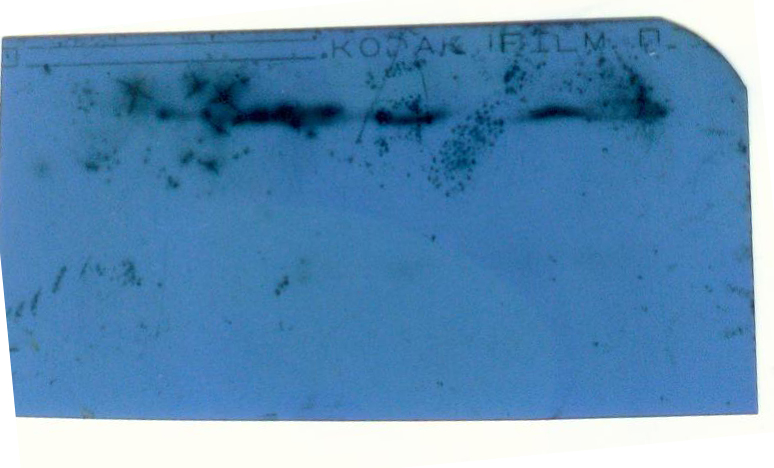


p53


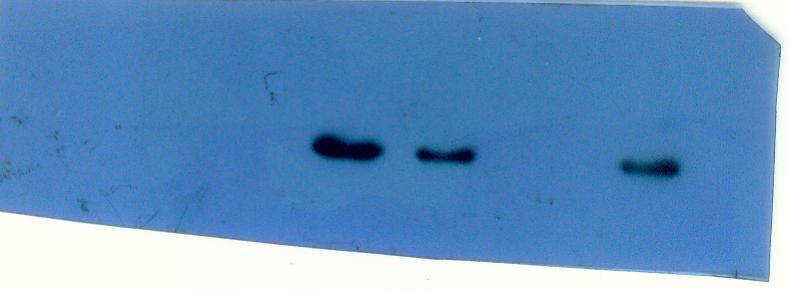


p-p53


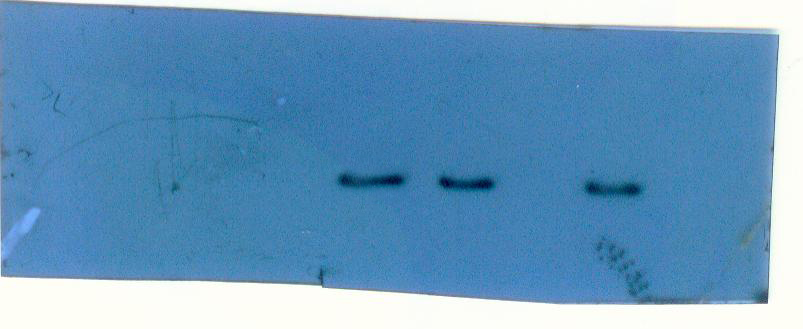


GAPDH


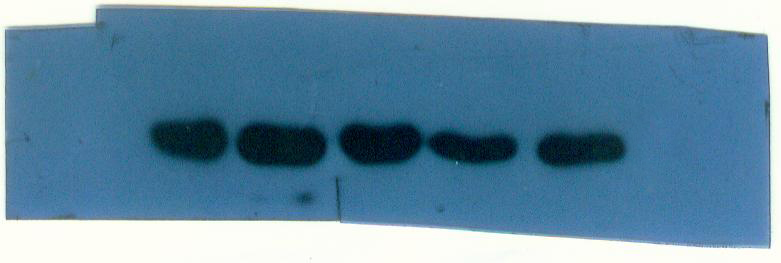


The red boxs are the Hep3B group and the blue boxs are the HepG2 group.
