# Supplementary figures and images for: Epigenetic identification of mitogen-activated protein kinase 10 as a functional tumor suppressor and clinical significance for hepatocellular carcinoma
Source: PeerJ. 2021 Feb 2;9:e10810. doi: 10.7717/peerj.10810 (PMC7863782; doi:10.7717/peerj.10810)

MARK 10

RT-PCR for MAPK 10

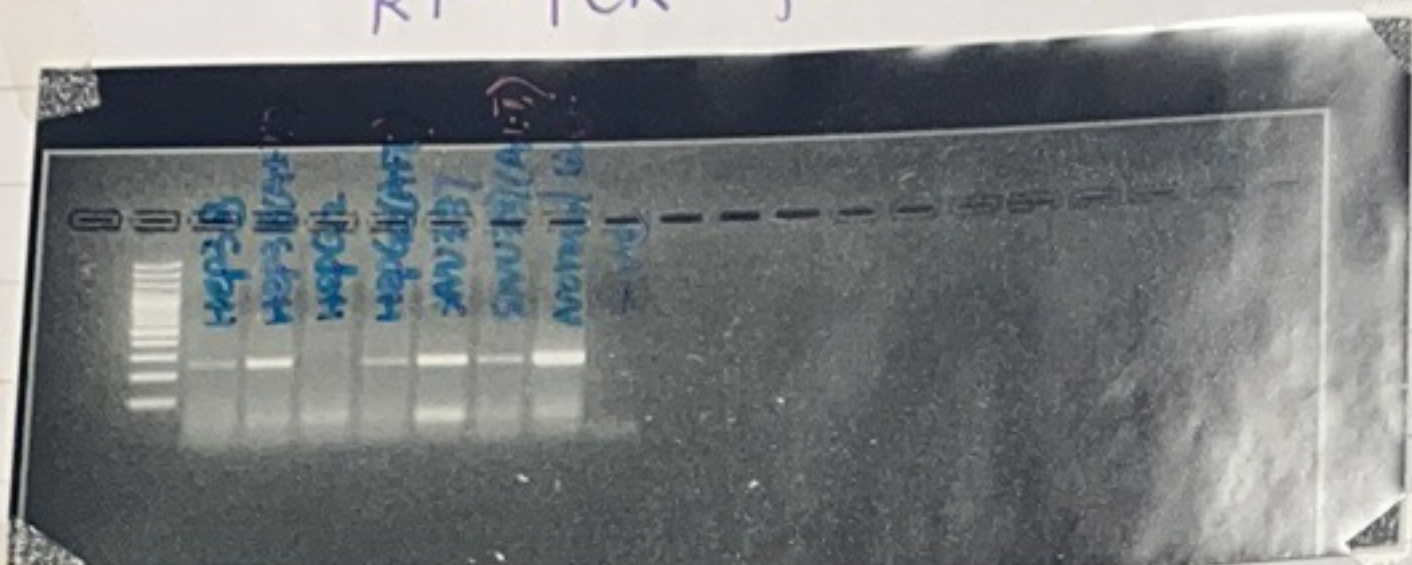

55°C      32 cycles  
2% crel

RT-PCR for GAPDH

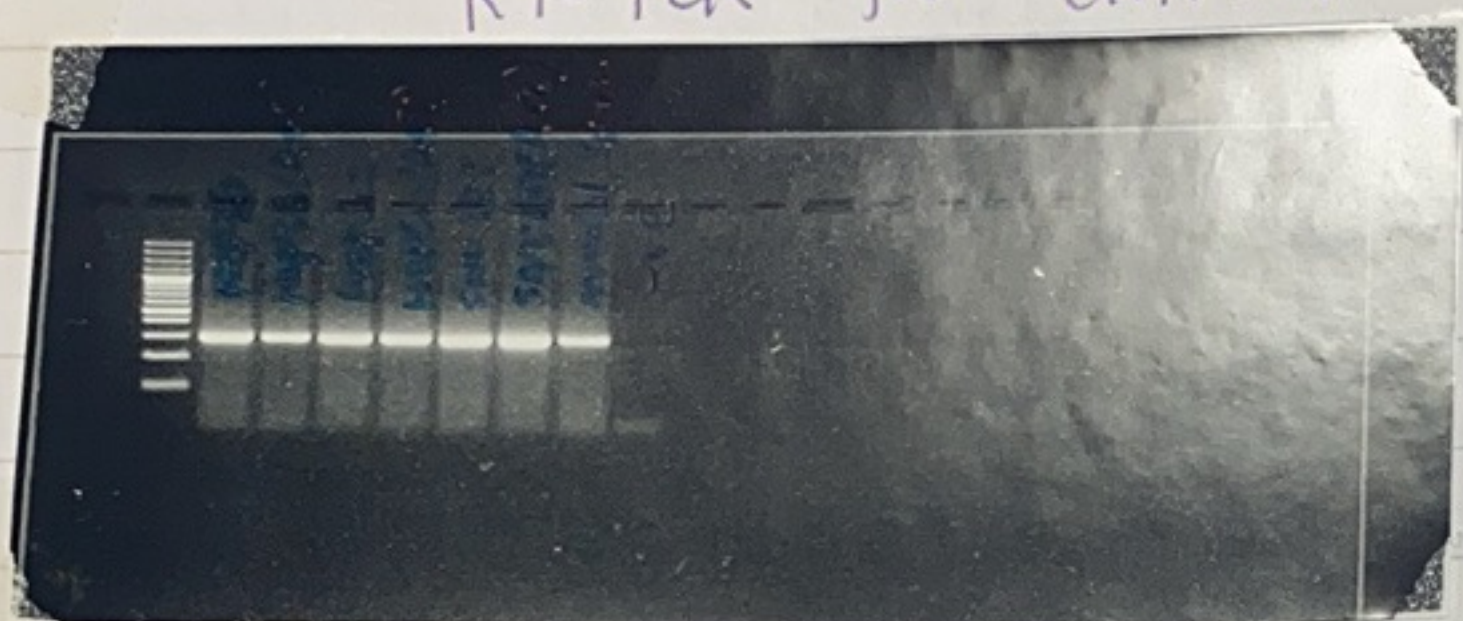

55°C      23 cycles  
27°C

m

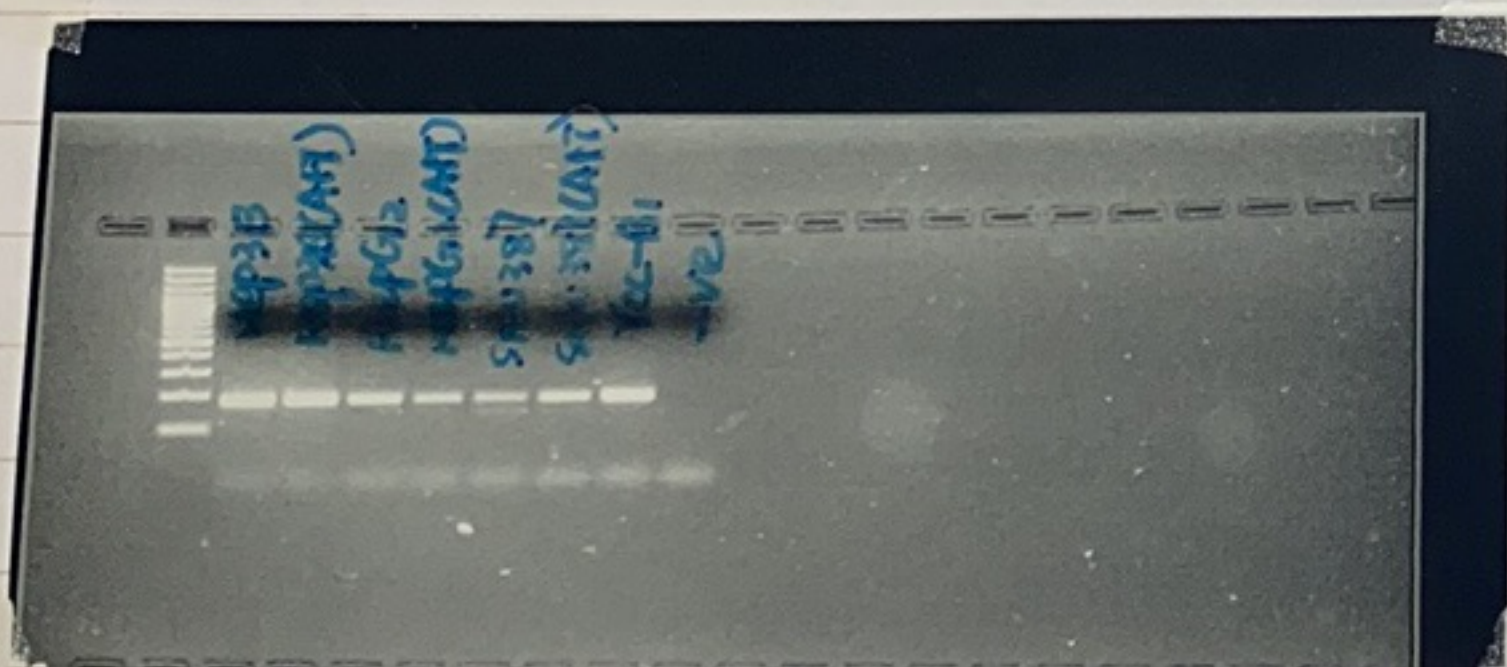

60°C      40 cycles  
2% Gel

V

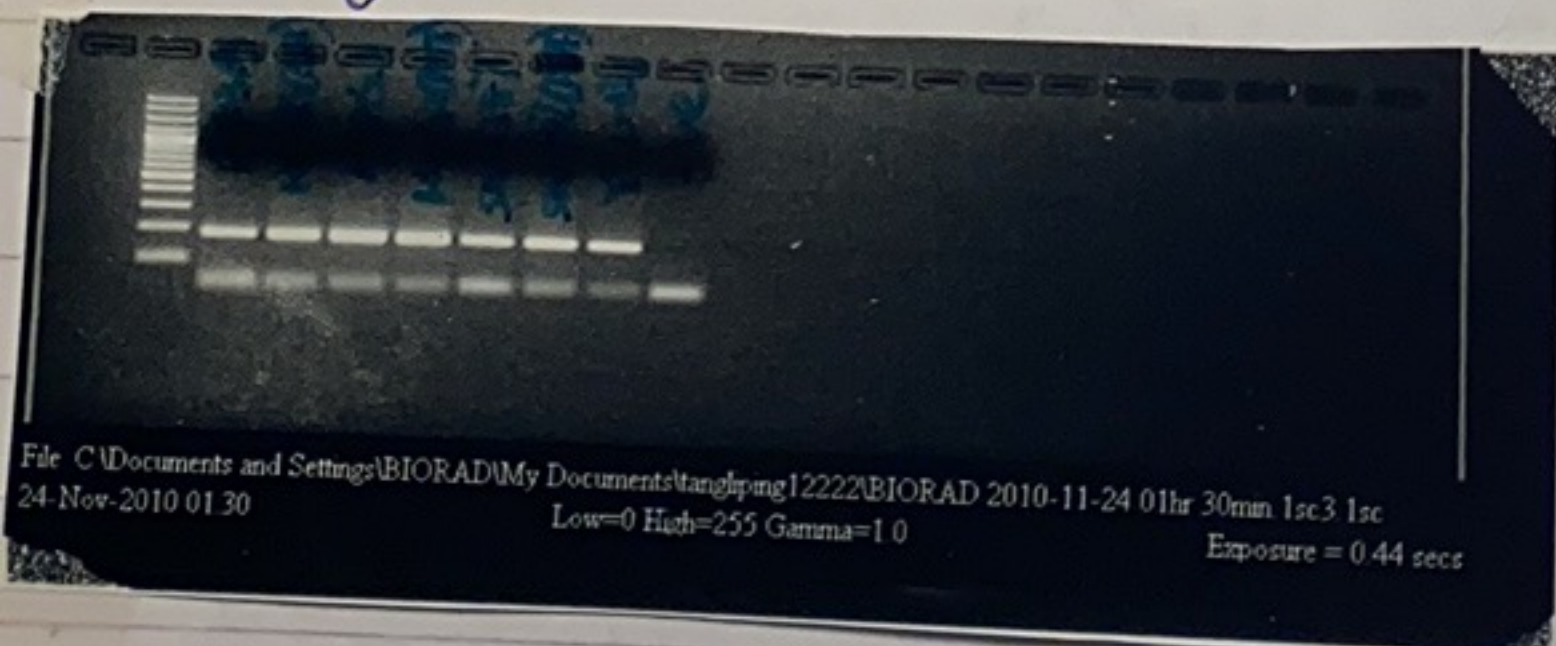

58°C      40 cycles  
2% Gel

Supplement: Supplemental Information 1 [file peerj-09-10810-s001.zip › 1.pdf]

MAPK 10 Msp

five

PCR

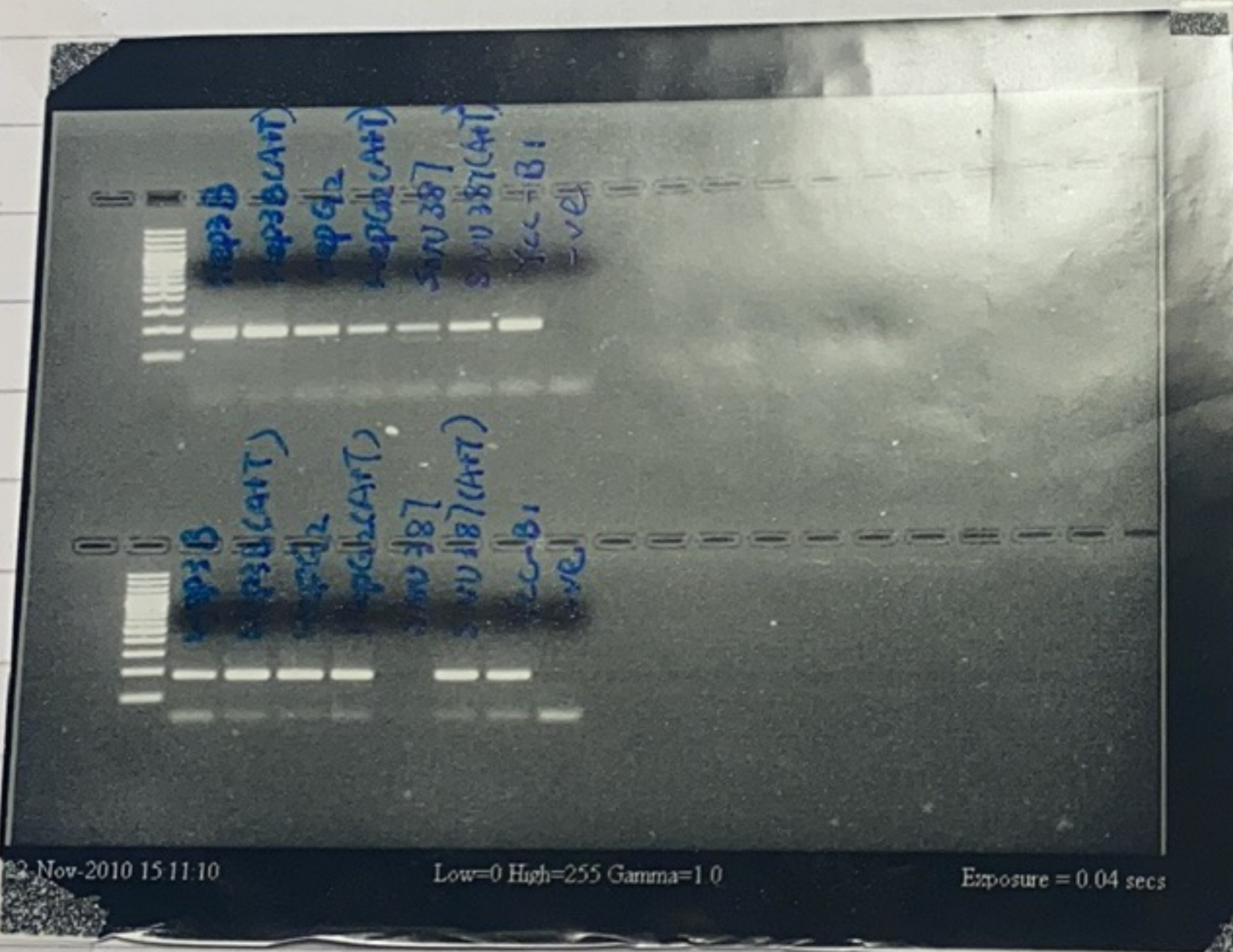

m: 60°C 40 cycles

U: 58°C 40 cycles

Supplement: Supplemental Information 1 [file peerj-09-10810-s001.zip › 2.pdf]

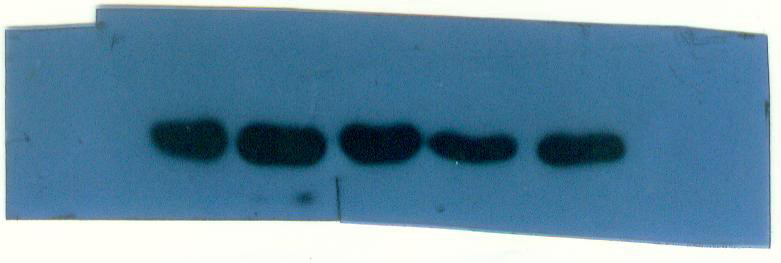

Supplement: Supplemental Information 1 [file peerj-09-10810-s001.zip › GAPDH.tif]

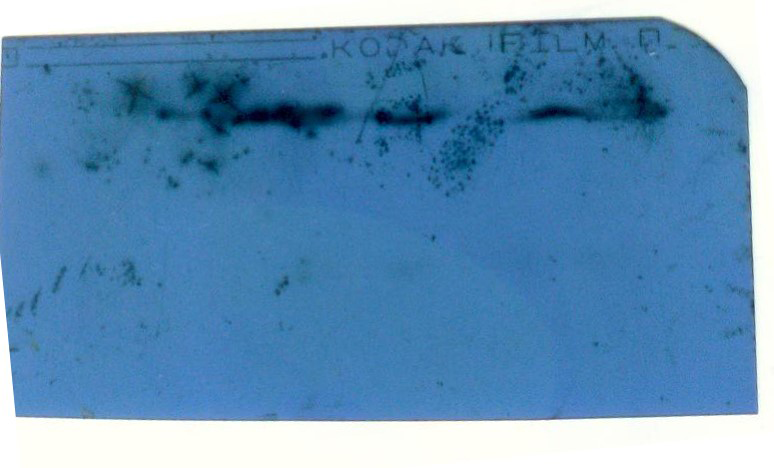

Supplement: Supplemental Information 1 [file peerj-09-10810-s001.zip › MAPK10.tif]

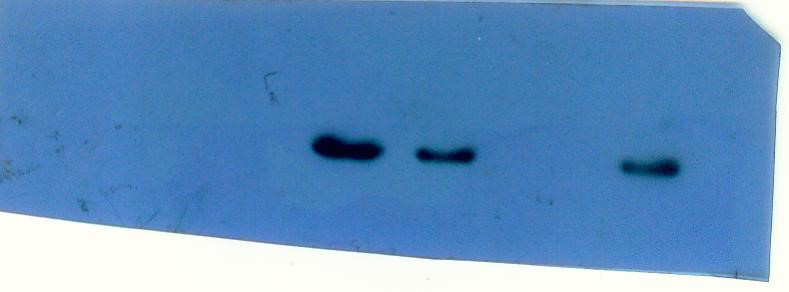

Supplement: Supplemental Information 1 [file peerj-09-10810-s001.zip › P53.tif]

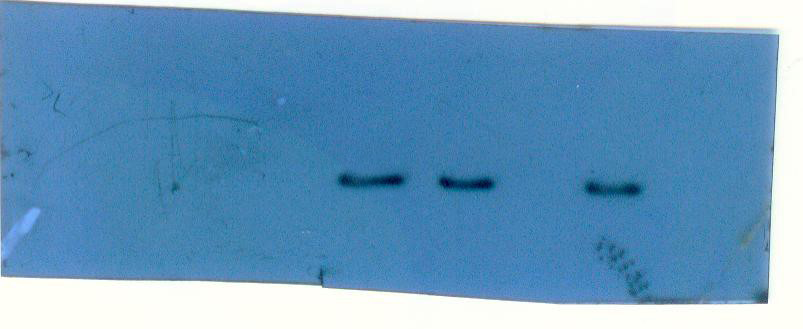

Supplement: Supplemental Information 1 [file peerj-09-10810-s001.zip › PP53.tif]
