# Supplementary material for: Epigenetic identification of mitogen-activated protein kinase 10 as a functional tumor suppressor and clinical significance for hepatocellular carcinoma
Source: PeerJ. 2021 Feb 2;9:e10810. doi: 10.7717/peerj.10810 (PMC7863782; doi:10.7717/peerj.10810)
Supplement: Supplemental Information 2 [file peerj-09-10810-s002.docx]

Supplementary Table 1. PCR primer sequences and reaction conditions

| PCR | Primers | Size (bp) | TA (℃) | PCR cycles |
| --- | --- | --- | --- | --- |
| RT-PCR |  |  |  |  |
| MAPK10 | F3: 5’-cagctctctaaattgactcag-3’ |  |  |  |
|  | RR3: 5’-ccaatgttggttcactgcag-3’ | 248 | 55 | 32 |
| GAPDH | 333: 5’-gatgaccttgcccacagcct-3’ |  |  |  |
|  | 355: 5’-atctctgcccctctgctga-3’ | 304 | 55 | 23 |
|  |  |  |  |  |
| Methylation-specific PCR (MSP) |  |  |  |  |
| Methylated | m3: 5’-cgagtagttttagcggttac-3’ | 160 | 60 | 40 |
|  | m5: 5’-aaaaccttctaacgcgaacga-3’ |  |  |  |
| Unmethylated | u3: 5’-tgtgagtagttttagtggttat-3’ | 163 | 58 | 40 |
|  | u5: 5’-caaaaccttctaacacaaacaa-3’ |  |  |  |

TA, annealing temperature.
